# Predictors of work engagement and burnout among rural nurses in Japan during COVID-19: a cross-sectional study

**DOI:** 10.1186/s12912-026-04616-w

**Published:** 2026-04-11

**Authors:** Yuri Kai, Maki Kanaoka, Aki Nozue, Sayaka Kato, Rinko Uchida, Ryuichiro Takeda, Yumiko Kinoshita

**Affiliations:** 1 Graduate School of Nursing Science, School of Nursing, Faculty of Medicine, University of Miyazaki, 5200 Kihara Kiyotake-cho, Miyazaki city, Miyazaki 889-1692 Japan; 2 Department of Nursing, Shimonoseki City University, 2-1-1 Daigakucho, Shimonoseki, Yamaguchi 751-8510 Japan; 3 Department of Nursing, Miyazaki Prefectural Nursing University, 3-5-1 Manabino, Miyazaki City, Miyazaki 880-0929 Japan; 4 Health and Safety Management Center, University of Miyazaki, 1-1 Gakuen Kibanadai-nishi, Miyazaki City, Miyazaki 889-2192 Japan

**Keywords:** Work engagement, Burnout, Job crafting, Resilience, Mental health, Nurses, COVID-19 pandemic, Rural hospitals

## Abstract

**Background:**

Work engagement and burnout are distinct psychological constructs, yet their coexistence under prolonged crisis conditions remains insufficiently examined, particularly in rural healthcare settings. This study investigated predictors of both work engagement and burnout among nurses in rural Japan during the later phase of the COVID-19 pandemic (July 2022).

**Methods:**

A cross-sectional paper-based survey was administered to all 560 nurses and midwives working at a rural university hospital. Missing data were minimal and handled using listwise deletion, resulting in a final analytical sample of 195 participants for regression analyses. Validated instruments assessed work engagement (UWES-3), burnout (BAT-J), job crafting (JCS-J), resilience (RS-14), and mental health (S-WHO-5-J). Hierarchical multiple linear regression analyses were conducted, with demographic variables entered first, followed by psychological variables and interaction terms to examine conditional associations with the COVID-19 care experience. Statistical significance was set at *p* < 0.05.

**Results:**

Work engagement was positively associated with job crafting (β = 0.31, *p* < 0.001) and resilience (β = 0.29, *p* < 0.001). The COVID-19 care experience moderated these relationships, weakening the association between job crafting and work engagement (β = −0.20, *p* = 0.003) while strengthening the association between mental health and work engagement (β = 0.21, *p* = 0.002), indicating indirect and conditional pathways rather than direct effects. Burnout was negatively associated with mental health (β = −0.46, *p* < 0.001) and resilience (β = −0.18, *p* = 0.014) and was higher among unmarried nurses (β = 0.13, *p* =0.047). No interaction effects were observed for burnout. Work engagement and burnout showed a weak inverse correlation (*r* = − 0.28).

**Conclusions:**

The psychological impact of COVID-19 varied by pandemic phase. In the later stage, the COVID-19 care experience was not directly associated with work engagement or burnout; however, it reconfigured how job and personal resources were linked to work engagement. Burnout was primarily linked to psychosocial factors rather than to the COVID-19 care experience. Sustaining nurse well-being during prolonged crises requires long-term organizational strategies that support job crafting, resilience, and accessible mental health care, with attention to regional and structural contexts, such as rural healthcare settings.

**Clinical trial number:**

Not applicable.

## Background

The prolonged COVID-19 pandemic has placed unprecedented psychological and occupational strain on nurses worldwide, intensifying concerns about workforce well-being and sustainability. Pandemic conditions have been associated with heightened emotional distress, moral tension, and burnout among frontline clinicians [1], and global evidence consistently demonstrates increased burnout among healthcare professionals, with intensive care unit (ICU) nurses at particularly high risk [2]. The pandemic has also adversely affected the mental and physical health of healthcare workers, potentially undermining work engagement [3]. Because nurses’ psychological functioning is closely linked to care quality, patient safety, and workforce retention, clarifying both negative and positive psychological outcomes during prolonged outbreaks remains a critical priority for sustaining healthcare systems.

Importantly, psychological responses during crises are not uniformly negative. Some nurses report adaptive reactions such as strengthened professional identity, enhanced emotional intelligence, and personal growth, suggesting that positive and negative processes can coexist under sustained adversity [4]. This duality aligns with positive psychology perspectives, which emphasize individuals’ capacity to mobilize strengths and motivation despite challenging conditions [5–7]. In this context, work engagement—a positive, fulfilling work-related state characterized by vigor, dedication, and absorption—has been recognized as a key indicator of sustainable work functioning [8]. In contrast, burnout reflects psychological ill-being arising from excessive demands and resource depletion. Contemporary scholarship emphasizes that work engagement and burnout are related yet distinct constructs that may coexist, particularly under prolonged stress conditions [9, 10]. Examining both outcomes concurrently is therefore essential to avoid oversimplifying nurses’ psychological experiences during extended crises.

Evidence from the COVID-19 pandemic further suggests that work engagement and burnout may develop through partially different psychological pathways. Caring for patients with (suspected) COVID-19 can strengthen meaning in work and professional identity, thereby heightening work engagement for some nurses [11–13]. At the same time, these duties may increase burnout through sustained workload, emotional burden, and infection-related concerns [2, 11–13]. This pattern implies that the COVID-19 care experience can activate both motivational and strain-related processes, underscoring the importance of examining predictors of work engagement and burnout simultaneously.

The Job Demands–Resources (JD–R) model provides a useful theoretical framework for understanding these dual processes [8, 14]. According to the model, job and personal resources foster work engagement through a motivational pathway, whereas excessive job demands contribute to burnout through a health-impairment pathway. Within this framework, resilience functions as a key personal resource that supports engagement and buffers stress, while job crafting represents proactive behaviors through which nurses modify aspects of their work to increase resources and improve person–job fit [15, 16]. Mental health represents proximal psychological strain within the health-impairment pathway and helps explain how accumulated job demands contribute to burnout.

Determinants of work engagement and burnout may also vary across phases of the pandemic and across healthcare settings. As infection-control protocols and vaccination coverage became established, some healthcare workers appeared to adapt psychologically, suggesting that predictors identified during the acute crisis phase may not fully generalize to later, more prolonged periods. Context is likewise critical. In Japan, rural hospitals face persistent structural constraints, including chronic staffing shortages, fewer specialized staff, limited access to back-up personnel, and higher workload per nurse. These challenges were further intensified during Japan’s seventh COVID-19 wave (July 2022), when several rural prefectures experienced severe medical strain. Despite these realities, evidence focusing on Japanese nurses—particularly those working in rural settings—and examining work engagement and burnout concurrently during prolonged outbreaks remains scarce. Prior studies have tended to address these outcomes separately or to focus primarily on urban institutions, limiting understanding of how engagement and burnout coexist and what predicts each outcome in rural Japanese hospitals [17–19].

Therefore, this study applied the Job Demands–Resources (JD–R) model to examine predictors of work engagement and burnout among nurses working in a rural Japanese university hospital during the seventh COVID-19 wave (July 2022). Specifically, we investigated whether job crafting and resilience (resources), mental health, and experience caring for COVID-19 patients were associated with work engagement and burnout, and whether these associations differed by outcome. By focusing on the later phase of the pandemic in a rural setting, this study provides evidence to inform context-sensitive strategies for sustaining the rural nursing workforce, including resource-oriented interventions and mental health support.

Guided by the JD–R model, this study tested the following a priori hypotheses to examine motivational and strain-related psychological processes among nurses during the prolonged phase of the pandemic:

### **H1**

Job resources and personal resources—specifically job crafting and resilience—are positively associated with work engagement, consistent with the motivational pathway of the JD–R model.

### **H2**

Poorer mental health is associated with higher burnout, reflecting the health-impairment pathway of the JD–R model.

### **H3**

Work engagement and burnout are inversely related yet remain conceptually and empirically distinct psychological constructs.

### **H4**

Experience caring for COVID-19 patients is associated with both work engagement and burnout; however, the nature and direction of these associations may differ, given prior evidence that COVID-related duties can elicit both stress-related and adaptive psychological responses.

## Methods

### Study design and setting

This cross-sectional study was conducted using self-administered questionnaires during the seventh wave of the COVID-19 pandemic in Japan (mid-2022). The study took place at University Hospital A, a rural university hospital located in a prefecture with a relatively small population and limited healthcare resources. The region is characterized by chronic nursing shortages, fewer specialized medical departments, limited availability of backup staff, and greater travel distances to tertiary referral centers—structural challenges commonly noted in rural Japanese healthcare systems. These contextual constraints were further intensified during the COVID-19 pandemic, particularly during the seventh wave, when regional healthcare capacity was substantially strained. During this period (summer 2022), a rapid surge in COVID-19 cases driven by the Omicron BA.5 variant placed substantial pressure on regional healthcare systems, particularly in rural areas [17, 20].

The study complied with the Strengthening the Reporting of Observational Studies in Epidemiology (STROBE) guidelines and the ethical principles of the Declaration of Helsinki. Ethical approval was obtained from the Institutional Review Board of the University of Miyazaki (approval number O-1126). Written informed consent was obtained from all participants. Confidentiality was strictly maintained, and permission for data collection in clinical areas was granted by nursing administration.

### Participants and sampling

The target population comprised all 560 nurses and midwives employed at University Hospital A as of July 2022. A census sampling approach was adopted, whereby questionnaires were distributed to the entire eligible workforce rather than to a convenience sample. Individuals in managerial positions (e.g., directors, department heads) were excluded to minimize potential bias arising from supervisory responsibilities, resulting in 536 eligible participants. All the nurses provided their informed consent to participate in the study before completing the questionnaire.

A total of 536 paper-based questionnaires were distributed. Of these, 203 were returned (response rate: 36.0%). After excluding questionnaires with missing data, 195 complete responses (valid response rate: 34.8%) were included in the analysis. Participation was voluntary and anonymous, and all participants provided written informed consent.

### Sample size estimation

Bentler and Chou [21] recommend a sample size of at least 10 times the number of observed variables. Based on this guideline, the final sample of 195 participants was considered sufficient for the planned statistical analyses. In addition, an a priori power analysis was conducted using G*Power 3.1 for multiple linear regression.

Assuming a medium effect size (f² = 0.15), a significance level of α = 0.05, statistical power (1 − β) = 0.80, and nine predictors, the required minimum sample size is approximately 118. The final sample size exceeded this requirement, indicating adequate statistical power for the planned analyses.

### Measures

#### Demographic characteristics

Participants reported demographic information (age, gender, marital status, educational background), along with COVID-19–related experiences (e.g., caring for patients with suspected or confirmed COVID-19) and work-related characteristics.

#### Work engagement

##### Utrecht work engagement scale-3 (UWES-3)

The UWES-3 assesses vitality, enthusiasm, and immersion using three items rated on a 7-point scale (0 = “not at all” to 6 = “always/every day”). Higher scores indicate greater work engagement. The Japanese version translated by Shimazu et al. [22] demonstrates strong reliability (Cronbach’s α = 0.88).

#### Burnout

##### Burnout assessment tool (BAT-J)

The BAT-J assesses burnout using two domains: core symptoms (tiredness, poor cognitive control, poor emotional control) and secondary symptoms (psychological distress, mental illness). The scale consists of 33 items, including core symptoms (23 items) and secondary symptoms (10 items). Items are rated on a 5-point scale (1 = “not at all” to 5 = “always”), with higher scores indicating more severe burnout. Reliability in previous research was high (Cronbach’s α = 0.96) [23].

#### Job crafting

##### Job crafting scale (JCS-J)

The JCS-J measures structural job resource enhancement, reduction of hindering job demands, interpersonal resource enhancement, and increased challenging job demands. The scale consists of 21 items, including structural job resource enhancement (5 items), reduction of hindering job demands (6 items), interpersonal resource enhancement (5 items), and increased challenging job demands (5 items). Each item is rated on a 5-point scale (1 = “not at all” to 5 = “often”). Cronbach’s α for subscales ranges from 0.67 to 0.90 [15, 16].

#### Resilience

##### Resilience scale short version (RS-14)

The RS-14 evaluates individual resilience and adaptability using 14 items rated on a 7-point scale (1 = “not applicable” to 7 = “very applicable”). Total scores range from 14 to 98, with higher scores indicating better resilience. Internal consistency is high (Cronbach’s α = 0.88) [24].

#### Mental health

##### WHO-5 (S-WHO-5-J)

The S-WHO-5-J consists of five items measuring well-being on a 4-point scale (0 = “never” to 3 = “always”). Higher scores indicate better mental health. It is a validated Japanese adaptation of the WHO-5 [25, 26].

### Data collection

Data were collected between July and August 2022, corresponding to Japan’s seventh COVID-19 wave. Paper-based, self-administered questionnaires were distributed to eligible nurses and midwives through the nursing administration office. Research staff provided instructions for survey completion, but they did not directly oversee responses to reduce response bias.

Participants completed the questionnaires voluntarily and returned them in sealed envelopes to designated collection boxes placed within the hospital. All submitted questionnaires were anonymized immediately upon receipt. Data were securely stored in password-protected files accessible only to the research team.

### Statistical analysis

Descriptive statistics were computed for participant characteristics and study variables. Internal consistency of each scale was assessed using Cronbach’s α coefficients. Work engagement (UWES-3) and burnout (BAT-J) were treated as dependent variables.

Descriptive analyses were conducted using all available cases (*N* = 195). Regression analyses were performed using complete cases after listwise deletion of missing data, resulting in a final analytical sample of 195 participants. As the proportion of missing data was small and showed no systematic pattern, listwise deletion was considered appropriate.

Hierarchical multiple linear regression analyses (forced-entry method) were conducted. Model 1 included control variables (sex, age group, marital status, educational background, job position, and the COVID-19 care experience). Model 2 added psychological predictors (job crafting, mental health, and resilience). Model 3 incorporated interaction terms to examine whether the COVID-19 care experience moderated these associations. Model fit was evaluated using F-change statistics, with statistical significance set at *p* < 0.05.

Continuous psychological predictors—job crafting (JCS-J), mental health (S-WHO-5), and resilience (RS-14)—were standardized to reduce multicollinearity. Interaction terms were created using standardized variables. Categorical variables were dummy-coded; marital status was coded as 0 = married and 1 = unmarried (including divorced), and the COVID-19 care experience as 0 = no experience and 1 = having cared for patients with confirmed or suspected COVID-19. Age group was treated as an ordinal variable and coded sequentially as 0 ≤ 30 years, 1 = 31–40 years, 2 = 41–50 years, and 3 ≥ 51 years. Educational background was coded as a binary variable (0 = below a bachelor’s degree, 1 = bachelor’s degree or higher). Job position was also coded as a binary variable (0 = staff nurse, 1 = managerial).

Assumptions of multicollinearity (VIF < 5), normality of residuals, and linearity were assessed and satisfied prior to analysis. All statistical analyses were conducted using Python (version 3.12.1) with the statsmodels package.

## Results

### Overview of descriptive results

Participants’ characteristics (Table 1), descriptive statistics and internal consistency of psychological measures (Table 2), and perceived pandemic-related stressors and support needs (Table 3) are summarized to provide contextual background.

**Table 1:** Background of participants 　(*N* = 195)

Variables		*n*	%
Sex	Men Women	14 181	7.2 92.8
Marriage	Married Not married (divorced)	94 101	48.2 51.8
Child	Yes No No response	75 119 1	38.5 61.0 0.5
Age (in years)	20–25 26–30 31–35 36–40 41–45 46–50 51–	38 27 27 31 28 17 27	19.5 13.8 13.8 15.9 14.4 8.7 13.8
Profession	Registered nurse Midwife	189 5	96.9 2.6
Academic background	Associate degree Bachelor’s degree Master’s degree No response	89 102 3 1	45.6 52.3 1.5 0.5
Years of experience (total)	0–5 6–10 11–15 16–20 21–25 26–30 31–35 36–	49 29 33 34 17 13 10 10	25.1 14.9 16.9 17.4 8.7 6.7 5.1 5.1
Position	Head nurse Assistant commissioned nurse Staff No response	8 25 158 4	4.1 12.8 81.0 2.1
Department	Internal medicine Surgical department Emergency, ICU, Operating Pediatric/Perinatal Psychiatry Radiology, Dentistry Anesthesiology Outpatient, patient support, and oncology department Other No response	50 35 15 29 12 3 20 18 7 6	25.6 17.9 7.7 14.9 6.2 1.5 10.3 9.2 3.6 3.1
Field of work	Acute phase Chronic phase End-of-life Other No response	103 45 6 29 12	52.8 23.1 3.1 14.9 6.2
Qualifications	Registered nurse Certified nurse No qualification Other No response	5 13 1 83 93	2.6 6.7 0.5 42.3 47.7
Experience of caring for COVID-19 patients	Yes No	68 127	34.9 65.1
Adviser (multiple responses)	Same period Younger people Seniors and supervisors No one	101 41 93 21	51.8 21.0 47.7 10.8
Willingness to continue in the nursing profession	Yes No No response	159 34 2	81.5 17.4 1.0

Note: Percentages were calculated based on the number of valid responses for each variable, while missing responses were excluded from percentage calculations. For items allowing multiple responses, percentages can sum to over 100%

**Table 2:** Descriptive statistics and reliability coefficients for each scale (*N* = 195)

Scale	Mean	SD	Cronbach’s α
Subscale			
**Work engagement (UWES-3)**			
Total average	2.73	1.22	0.88
**Vitality**	2.54	1.25	0.95
**Enthusiasm**	2.52	1.46	0.94
**Immersing oneself**	3.13	1.35	0.93
**Job crafting (JCS-J)**			
Total amount	52.5	10.5	0.61
Improvement of structural work resources	14.8	3.80	0.72
Reduction in the level of demand for disruptive work	15.4	4.60	0.76
Improved social work resources	11.4	3.00	0.75
Heightened demand for challenging work	10.9	3.90	0.70
**Burnout assessment (BAT-J)**			
Total average	2.7	0.59	0.91
Core symptom	2.8	0.56	0.91
Feeling of exhaustion	3.6	0.78	0.92
Psychological distance	2.3	0.72	0.92
Cognitive control malfunction	2.4	0.72	0.92
Malfunction of emotional control	2.2	0.71	0.93
Secondary symptoms	2.6	0.80	0.91
Psychological distress	2.8	0.86	0.91
Mental and physical disorder	2.3	0.92	0.92
**Resilience (RS-14)**	62.3	11.5	0.92
**WHO-5 Well-Being Index (S-WHO-5-J)**	7.2	2.9	0.90

Table 1 presents the demographic and occupational characteristics of the participants. Of the 195 valid respondents (response rate: 34.8%), 189 (96.9%) were nurses, 181 (92.8%) were women, 65 (33.0%) were aged 20–30 years, and 68 (34.9%) reported experience caring for confirmed or suspected COVID-19 patients. These demographic variables were included as background covariates in subsequent regression analyses.

Table 2 summarizes the descriptive statistics and reliability indices (Cronbach’s α) for all psychological variables, including work engagement (UWES-3), burnout (BAT-J), job crafting (JCS-J), resilience (RS-14), and mental health (WHO-5). All scales demonstrated acceptable internal consistency, supporting their use in multivariable analyses.

Table 3 describes participants’ perceived pandemic-related stressors and support needs. These items reflect job demands and job resources within the JD–R framework and informed the selection and interpretation of predictors included in the regression models.

**Table 3:** Impact of COVID-19 pandemic and important support details (*N* = 195)

Impact of the COVID-19 pandemic*	**Mean (SD)**
I worry that I might infect my family. I worry that I might infect my co-workers. I worry about infecting co-workers by allowing family members to infect themselves with COVID-19. I worry that my family will infect me. I find it difficult to support patient decision-making. I think the visitation restrictions are affecting the patients/families.	2.3 (1.2) 2.4 (1.2) 3.0 (1.3) 2.9 (1.3) 2.1 (0.96) 1.3 (0.57)
**Importance of support during the COVID-19 pandemic (Priority 1–3)**	**Mean (** ***SD*** **)**
Counselling Gratitude or respect Financial rewards Educational resources for infection prevention Childcare support More staff members Reduction of workload	0.6 (1.8) 1.1 (2.4) 2.0 (2.7) 0.92 (2.2) 0.72 (2.0) 6.3 (3.5) 5.9 (3.2)

Note: Impact of the COVID-19 infection was rated on a Likert scale of 1 (very much agree) to 5 (not at all agree), and the mean score was calculated. Importance of support during the COVID-19 pandemic: The order of importance of support was rated from 1–3, and the average score calculated was converted into 10 points for “1”, 5 points for number “2”, and 3 points for number “3”

### Factors associated with work engagement

Hierarchical multiple regression analyses were conducted to identify predictors of work engagement (UWES-3). In the main-effects model, job crafting (β = 0.31, *p* <0.001) and resilience (β = 0.29, *p* <0.01) were positively associated with work engagement, whereas mental health did not show a significant main effect. The model explained a substantial proportion of variance in work engagement.

**Fig. 1:**
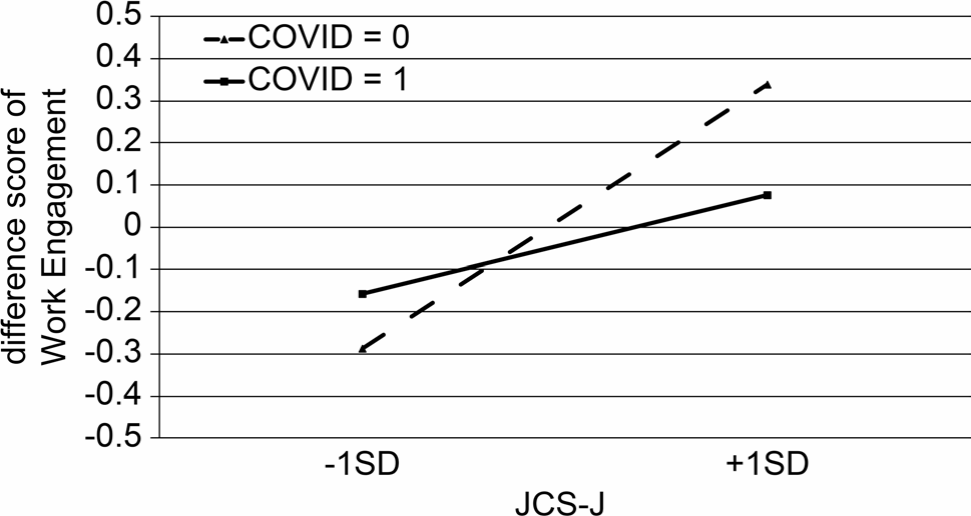
Interaction effect of job crafting and COVID-19 care experience on work engagement

**Fig. 2:**
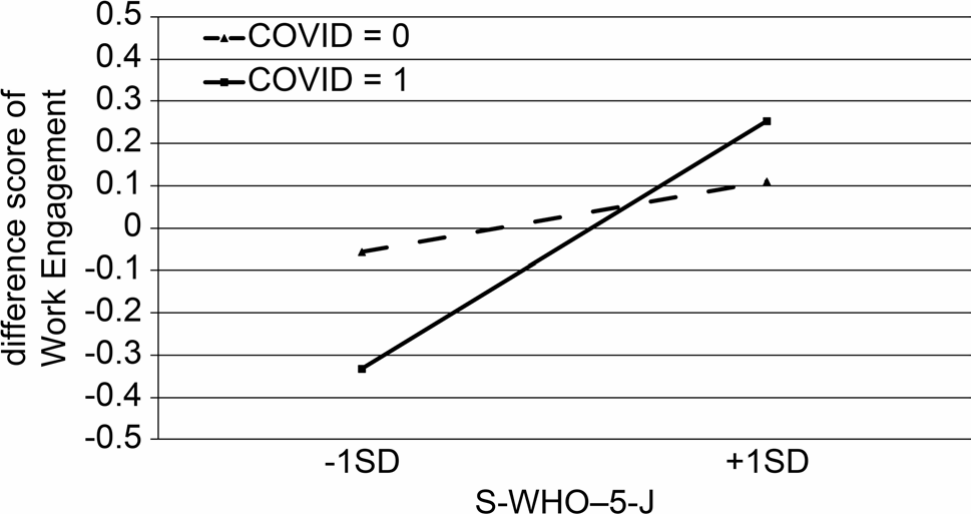
Interaction effect of mental health and COVID-19 care experience on work engagement

Interaction analyses (Model 3) identified two significant moderating effects involving the COVID-19 care experience. First, experience caring for COVID-19 patients weakened the positive association between job crafting and work engagement (β = −0.20, *p* = 0.003). Second, the COVID-19 care experience strengthened the association between mental health and work engagement (β = 0.21, *p* = 0.002). These interaction effects are illustrated in Figs. 1 and 2. Overall, the associations between psychological resources and work engagement differed based on nurses’ exposure to COVID-19 patient care. The results of the hierarchical regression analysis predicting work engagement are shown in Table 4.

**Table 4:** Hierarchical regression analysis predicting work engagement with COVID-19 care experience as moderator (*N* = 195)

	Model 1	Model 2	Model 3
Sex	0.56	0.73	0.73
Age group	1.18	0.50	0.75
Married / Unmarried	− 0.74	− 0.11	0.38
Education (Below / Higher)	− 0.01	0.60	0.84
Position	1.09	0.94	1.15
COVID-19 Care	− 0.10	-1.33	-1.07
JCS-J Total		4.78***	4.79***
S-WHO-5-J Total		1.21	1.23
RS-14 Total		3.59***	3.80***
COVID-19 Care × JCS-J Total			-3.00**
COVID-19 Care × S-WHO-5-J Total			3.13**
COVID-19 Care × RS-14 Total			− 0.90
R²	0.05	0.31	0.37
Adjusted R²	0.01	0.28	0.33
R² change	0.05	0.27 **	0.06 **
F	1.43	9.11***	8.84***

* *p* < 0.05, ** *p* < 0.01, *** *p* < 0.001

Notes. Marital status: 0 = married, 1 = unmarried (including divorced); COVID-19 care experience: 0 = none, 1 = experience caring for confirmed or suspected COVID-19 patients: Age group (ordinal): 0 ≤ 30, 1 = 31–40, 2 = 41–50, 3 ≥ 51 years; Educational background: 0 = below a bachelor’s degree, 1 = bachelor’s degree or higher

Job position: 0 = staff nurse, 1 = managerial

Values represent t-statistics. Standardized regression coefficients (β) are reported in the text

### Factors associated with burnout

For burnout (BAT-J), mental health was the strongest predictor (β = −0.46, *p* < 0.001), followed by resilience (β = −0.18, *p* = 0.014). In addition, unmarried nurses reported significantly higher burnout levels than married nurses (β = 0.13, *p* = 0.047). Together, these variables explained a substantial proportion of variance in burnout.

No interaction terms involving the COVID-19 care experience reached statistical significance for burnout (p-values ranged from 0.11 to 0.47), suggesting that the associations between psychological factors and burnout were not moderated by the COVID-19 care experience. Table 5 presents the hierarchical regression analysis predicting burnout.

**Table 5:** Hierarchical regression analysis predicting burnout with COVID-19 care experience as a moderator (*N* = 195)

	Model 1	Model 2	Model 3
Sex	0.49	1.30	1.11
Age group	-1.45	-1.24	-1.16
Married / Unmarried	2.77**	1.98*	2.00*
Education (Below / Higher)	0.17	− 0.04	− 0.22
Position	− 0.19	− 0.84	-1.04
COVID-19 Care	− 0.47	0.04	− 0.02
JCS-J Total		1.17	1.17
S-WHO-5-J Total		-7.00***	-6.90***
RS-14 Total		-2.45*	-2.48*
COVID-19 Care × JCS-J Total			− 0.73
COVID-19 Care × S-WHO-5-J Total			-1.61
COVID-19 Care × RS-14 Total			0.98
R²	0.10	0.39	0.40
Adjusted R²	0.07	0.36	0.36
R² change	0.10	0.29***	0.01
F	3.46**	12.8***	9.94 ***

* *p* < 0.05, ** *p* < 0.01, *** *p* < 0.001

Marital status: 0 = married, 1 = unmarried (including divorced); COVID-19 care experience: 0 = none, 1 = experience caring for confirmed or suspected COVID-19 patients; Age group (ordinal): 0 ≤ 30, 1 = 31–40, 2 = 41–50, 3 ≥ 51 years; Educational background: 0 = below a bachelor’s degree, 1 = bachelor’s degree or higher. Job position: 0 = staff nurse, 1 = managerial

Values represent t-statistics. Standardized regression coefficients (β) are reported in the text

### Relationship between work engagement and burnout

Correlation analysis revealed a weak inverse association between work engagement and burnout (*r* = −0.28). This modest correlation supports the interpretation that work engagement and burnout are related yet empirically distinct psychological constructs rather than opposite ends of a single continuum in this sample. Pearson correlation coefficients among the main study variables are presented in Table 6.

**Table 6:** Pearson correlation matrix among study variables (*N* = 195)

	UWES-3	JCS-J	BAT-J	RS-14	S-WHO-5-J
UWES-3					
JCS-J	0.46				
BAT-J	− 0.28	− 0.15			
RS-14	0.46	0.40	− 0.45		
S-WHO-5-J	0.29	0.24	− 0.55	0.44	

UWES-3 = Utrecht Work Engagement Scale-3 (Japanese version); BAT-J = Burnout Assessment Tool (Japanese version); JCS-J = Job Crafting Scale (Japanese version); RS-14 = Resilience Scale, short version (14-item, Japanese version); S-WHO-5-J = WHO-5 Well-Being Index, short version (Japanese version)

Pearson correlation coefficients

All correlations were statistically significant at *p* < 0.001

### Hypothesis testing

In accordance with the study aims, we formally tested the four hypotheses derived from the JD–R model.

#### H1

Job resources and personal resources—specifically job crafting and resilience—are positively associated with work engagement, consistent with the motivational pathway of the JD–R model.

Regression analyses confirmed that job crafting (β = 0.31, *p* < 0.001) and resilience (β = 0.29, *p* < 0.001) were significant positive predictors of work engagement. These findings support the JD–R model’s motivational pathway [8, 14]. H1 is therefore supported.

#### H2

Mental health status is negatively associated with burnout, reflecting the health-impairment pathway of the JD–R model.

Burnout was significantly and negatively predicted by mental health (β = −0.46, *p* < 0.001). Resilience and marital status also showed significant associations, indicating that burnout was primarily shaped by psychological well-being and personal resources. These results are consistent with the JD–R model’s health-impairment pathway [8, 14]. H2 is therefore supported.

#### H3

Work engagement and burnout are inversely related yet remain conceptually and empirically distinct psychological constructs.

A weak negative correlation was observed between work engagement and burnout (*r* = − 0.28). Combined with their distinct regression predictors, this finding supports the conceptualization that engagement and burnout are related yet separate psychological processes rather than opposite ends of a single continuum [9, 10]. H3 is therefore supported.

#### H4

Experience caring for COVID-19 patients is associated with work engagement and burnout, with the nature of these associations potentially differing across outcomes.

No significant main effect was found between the COVID-19 care experience and work engagement or burnout. However, significant interaction effects were observed for work engagement, indicating that the COVID-19 care experience moderates the relationships between psychological resources and work engagement. Specifically, the associations of job crafting and psychological well-being with work engagement differed based on whether nurses had experience in caring for COVID-19 patients.

In contrast, no significant interaction effects were found for burnout, suggesting that the COVID-19 care experience influenced burnout neither directly nor indirectly in this model. Taken together, H4 was partially supported, as the COVID-19 care experience showed a context-dependent moderating effect on work engagement but not on burnout.

## Discussion

This study examined the determinants of work engagement and burnout among nurses working in a rural Japanese university hospital during the peak of Japan’s seventh COVID-19 wave (July–August 2022). By analyzing work engagement and burnout simultaneously within the JD–R framework, the study provides new insights into how positive and negative psychological states diverge under prolonged public health emergencies. Importantly, all interpretations are framed as associative and context-dependent, given the cross-sectional nature of the study.

### Determinants of work engagement: job and personal resources

Work engagement was significantly associated with job crafting and resilience, supporting H1. These findings align with the JD–R model, in which job and personal resources activate motivational processes that foster energy, dedication, and fulfillment [8, 14]. Job crafting emerged as the strongest predictor. This is consistent with prior research demonstrating that proactively modifying tasks, seeking additional resources, or redefining work meaning enhances autonomy, meaning, and engagement [15, 16]. Resilience also predicted work engagement, in line with evidence that resilient nurses regulate emotions more effectively and maintain motivation under adversity [6, 17].

Importantly, hierarchical regression analysis further revealed that nurses’ experience with COVID-19 patient care moderated the association between work engagement and job and personal resources. Specifically, significant interaction effects were observed for job crafting and mental health, indicating that the strength and direction of these associations differed based on whether nurses had the COVID-19 care experience. These findings suggest that the COVID-19 care experience does not directly influence work engagement but instead conditions how available resources are translated into engagement.

In contrast, resilience showed a robust main effect but not a significant interaction effect, indicating that its positive association with work engagement is consistent regardless of nurses’ COVID-19 care experience. Moreover, this experience alone was not directly associated with work engagement, suggesting that by the seventh wave of the pandemic, COVID-related duties should be interpreted as contextual job demands rather than independent motivational drivers. Taken together, these results reinforce that work engagement is primarily shaped by resource-driven motivational pathways and that the impact of extraordinary job demands, such as COVID-19 care, depends on how individual and job resources are mobilized rather than on exposure itself.

### Determinants of burnout: psychological strain

Burnout was predicted only by mental health status, supporting H2. This finding is consistent with prior systematic reviews and later-phase pandemic studies reporting that psychological distress and depressive symptoms are the strongest determinants of burnout among nurses [2, 19]. Consistent with this trend, the COVID-19 care experience did not predict burnout, further supporting H4’s expectation that associations might differ from early-pandemic patterns [11–13, 17–19]. As the pandemic progressed, improvements in infection-control knowledge, teamwork, procedural confidence, and the routinization of COVID-related tasks may have attenuated fear-driven emotional overload, leaving burnout more strongly linked to enduring psychological strain than to pandemic-specific exposures.

### Coexistence of engagement and burnout

The weak negative correlation between work engagement and burnout supported H3, indicating that the constructs are related yet distinct. This is consistent with prior JD–R research demonstrating that work engagement and burnout stem from separate motivational and health-impairment pathways [8, 14]. Meaning-making under adversity may also explain this coexistence: challenging clinical work can strengthen identity and purpose even as emotional exhaustion accumulates [6, 9]. These findings underscore the importance of evaluating both constructs concurrently to achieve a more nuanced understanding of nurses’ psychological responses during health-system crises.

### Indirect and conditional influence of the COVID-19 care experience on work engagement through job crafting

Experience caring for COVID-19 patients did not show a significant *direct* association with either work engagement or burnout. However, a more nuanced pattern emerged when interaction effects were considered, suggesting that the influence of the COVID-19 care experience operates through indirect and conditional pathways, particularly via job crafting and mental health.

Consistent with differences in the psychological impact of COVID-19 across various pandemic stages, these findings reflect conditions characteristic of a later, more stabilized phase by the seventh wave. As clinical workflows, infection-control procedures, and treatment protocols became routinized, the perceived novelty, unpredictability, and threat associated with COVID-19 care diminished. Previous studies have shown that healthcare workers gradually developed psychological adaptation during later pandemic stages, characterized by the normalization of COVID-related tasks, increased confidence in infection-prevention measures, and reduced fear of infection [11, 12]. Accordingly, regional infection severity is interpreted as background system strain rather than a direct explanatory factor for work engagement levels.

Importantly, although the COVID-19 care experience did not directly increase work engagement, it was associated with qualitative changes in how psychological resources functioned. Specifically, it *weakened* the positive association between job crafting and work engagement. This finding suggests that among nurses with the COVID-19 care experience, proactive job redesign may have become less discretionary and more necessity-driven, reflecting adaptive maintenance of functioning under constrained conditions rather than optional motivation enhancement. This interpretation aligns with JD–R theory, which proposes that job crafting can serve both motivational and compensatory functions depending on contextual demands.

At the same time, the COVID-19 care experience strengthened the association between mental health and work engagement, indicating that for nurses exposed to prolonged high-demand conditions, psychological well-being became a prerequisite for sustaining engagement. Under prolonged stress conditions, job-related strategies alone may be insufficient if positive mental health is not maintained. This finding highlights a shift from task-focused to person-centered determinants of engagement in the later pandemic stage.

Taken together, these interaction effects suggest that the COVID-19 care experience did not simply “lose relevance,” but rather reconfigured the pathways leading to work engagement, supporting the conceptualization of work engagement and burnout as dynamic, context-sensitive outcomes.

### Rural healthcare context

Rural hospitals in Japan face chronic staffing shortages, limited specialist availability, and fewer backup personnel—conditions that were further intensified during the seventh COVID-19 wave [17, 18]. These structural constraints likely increased job demands and heightened vulnerability to burnout. In this study, burnout appeared to reflect the cumulative effects of long-standing workload pressures and reduced mental health rather than pandemic-specific exposures [2, 19]. Conversely, strong community bonds and close patient–provider relationships may enhance job resources such as perceived recognition, sense of impact, and work meaningfulness. These contextual features may help explain why rural nurses relied more heavily on stable personal and job resources—such as resilience and job crafting—rather than situational demands like the COVID-19 care experience to maintain work engagement during the later pandemic stage [15–19, 24].

Financial incentives did not predict work engagement in this sample, in contrast to urban studies reporting that monetary rewards support engagement [17]. This contrast provides the empirical rationale for advocating regionally tailored workforce strategies, suggesting that in rural settings, nursing support should prioritize staffing reinforcement, workload redistribution, protected recovery time, and long-term workforce investment rather than relying primarily on metropolitan-style financial incentives. These aspects are consistent with the Job Demands–Resources model, which posits that social and relational resources foster work engagement and meaningfulness by enhancing available job resources [8, 14].

### Strengths and limitations

#### Strengths

This study has several notable strengths. First, it simultaneously examined work engagement and burnout within the JD–R framework, enabling a comprehensive understanding of how positive (engagement) and negative (burnout) psychological states can coexist during prolonged public health emergencies [8, 14].

Second, the survey was conducted during the peak of Japan’s seventh COVID-19 wave, providing rare and contextually grounded insights into nurses’ psychological adaptation during a later phase of the pandemic—an understudied period in which COVID-specific stressors may have diminished while chronic job demands persisted [11–13, 17–19].

Third, by focusing on nurses working in a rural university hospital—an underrepresented population in the COVID-19 literature—this study highlights how distinctive structural conditions, such as chronic staffing shortages, limited specialist availability, and strong community ties, shape work engagement and burnout differently from urban healthcare settings [17–19].

Finally, the use of well-validated instruments (UWES-3, BAT-J, JCS-J, RS-14, S-WHO-5-J) [22–26], together with the inclusion of crisis-specific variables such as the COVID-19 care experience, enabled a rigorous and multidimensional assessment of factors influencing work engagement and burnout under high-strain conditions.

#### Limitations

Several limitations should be considered when interpreting the findings. First, the study was conducted at a single rural university hospital, which may limit the generalizability of the results to other regions or healthcare institutions with different organizational structures.

Second, the cross-sectional design precludes causal inference regarding the relationships among job resources, personal resources, mental health, and psychological outcomes [8, 14].

Third, although the response rate (34.8%) is reasonable given the crisis context, non-response bias cannot be ruled out, as nurses experiencing higher workloads or psychological distress may have been less likely to participate.

Fourth, the reliance on self-reported questionnaires introduces the possibility of recall bias and social desirability effects.

Fifth, data collection occurred specifically during Japan’s seventh COVID-19 wave—a period characterized by high medical strain alongside increasing psychological adaptation among healthcare workers [11–13, 17–19]. Consequently, the determinants of work engagement and burnout identified in this study may differ from those observed during earlier or later pandemic phases, or in non-pandemic contexts.

Sixth, rural hospitals possess unique structural characteristics, including chronic staffing shortages, limited backup capacity, and constrained specialist availability [17–19]. These contextual features may shape engagement and burnout differently than in metropolitan settings, potentially limiting transferability.

Finally, this study did not assess organizational-level factors such as leadership style, team climate, emotional labor, or the availability of formal support systems, all of which may influence work engagement and burnout and should be examined in future research.

Despite these limitations, the study offers meaningful theoretical and practical contributions by clarifying how distinct JD–R pathways shape work engagement and burnout and by providing crisis- and context-specific evidence to inform interventions aimed at supporting nurse well-being during future public health emergencies.

### Implications for practice and policy

Based on the study findings, several practice- and policy-level implications emerge for strengthening nurse well-being during prolonged public health emergencies, particularly in rural healthcare settings.

#### Promote job crafting interventions

Because job crafting was the strongest predictor of work engagement, structured job crafting programs—such as workshops on task redesign, resource seeking, or reframing work meaning—should be incorporated into staff development and continuing education [15, 16]. Facilitating proactive resource-building may enhance autonomy, engagement, and adaptability during crises.

#### Strengthen resilience-building programs

Resilience emerged as a key personal resource supporting engagement. Hospitals should implement resilience-enhancing initiatives, including mindfulness-based programs, peer-support systems, reflective practice sessions, and training in adaptive coping [6, 24]. Such interventions can help nurses maintain motivation under prolonged strain.

#### Recognize mental health support to reduce burnout

Given the strong association between burnout and poor mental health, proactive mental health screening, confidential psychological services, and accessible counseling resources should be institutional priorities [2, 19, 25, 26]. Embedding routine check-ins and early support pathways can reduce psychological distress before it escalates into burnout.

#### Tailor workforce strategies to rural conditions

Findings highlight the need for region-specific workforce policies. Rural hospitals—characterized by chronic staffing shortages, limited specialist availability, and reduced backup capacity—require structural interventions such as reinforcement staffing, flexible float pools, equitable workload redistribution, and investments in long-term workforce capacity [17–19]. In these contexts, metropolitan-style financial incentives may be insufficient without parallel structural support.

#### Recognize and support frontline clinical roles

Although COVID-19 caregiving did not directly predict engagement or burnout in this late pandemic phase, recognition of frontline work remains essential. Organizational acknowledgment, visible appreciation initiatives, and community-based recognition can strengthen professional identity and meaning, thereby supporting engagement indirectly through adaptive pathways such as job crafting and psychological well-being [11–13].

### Future research directions

Future research should build on these findings in several ways. First, longitudinal studies are needed to examine how work engagement and burnout evolve across different phases of a public health crisis and to assess whether the temporal shift observed during the seventh COVID-19 wave reflects broader patterns of psychological adaptation [11–13, 17–19].

Second, mediation and moderation analyses using larger, multi-site samples could clarify the mechanisms through which job and personal resources influence engagement and burnout. For example, resilience may mediate the relationship between job crafting and work engagement, while mental health may mediate or moderate the pathways leading to burnout [8, 14–16, 24].

Third, comparative studies between rural and urban hospitals are required to identify region-specific determinants of work engagement and burnout. Such comparisons would help determine whether the structural constraints and community-based resources characteristic of rural healthcare settings produce distinct psychological responses or resource patterns [17–19].

Fourth, qualitative research—including interviews, focus groups, and narrative analyses—could enrich the understanding of how nurses construct meaning, professional identity, and psychological growth through crisis caregiving, as well as how they navigate prolonged periods of uncertainty and strain [4, 6, 11, 12].

Finally, future studies should incorporate organizational-level variables, such as leadership style, team climate, emotional labor, and availability of formal support systems, to provide a more comprehensive understanding of the multilevel factors influencing nurse well-being [8, 14].

## Conclusions

Using the JD–R framework, this study examined determinants of work engagement and burnout among nurses at a rural Japanese university hospital during the peak of Japan’s seventh COVID-19 wave. Experience caring for COVID-19 patients did not directly predict work engagement or burnout; instead, its association with work engagement emerged through indirect pathways involving proactive job crafting behaviors and psychological well-being. This finding suggests that, during the later phase of the pandemic, the psychological impact of COVID-19 was characterized less by infection-specific stressors and more by adaptive, resource-based mechanisms as nurses adjusted to prolonged crisis conditions. Work engagement was primarily shaped by job and personal resources. Job crafting emerged as the strongest predictor, suggesting that nurses who proactively modify their tasks or seek resources are better able to sustain motivation under prolonged strain. Resilience also contributed significantly, underscoring the importance of psychological adaptability in maintaining engagement during extended public health emergencies.

In contrast, burnout was predicted by mental health status and marital status, highlighting the central role of psychological strain and social support in shaping exhaustion during the later pandemic stage. Chronic job demands—such as staffing shortages, emotional burden, and insufficient recovery time—may overwhelm individual coping capacities, leading to burnout regardless of engagement levels. These findings indicate that interventions aimed solely at enhancing engagement are insufficient to prevent burnout without robust mental health support.

The modest negative correlation between work engagement and burnout further supports the view that these constructs represent related but distinct psychological processes with different antecedents. Nurses may remain motivated and dedicated while simultaneously experiencing emotional fatigue, particularly in high-demand rural hospital settings. Effective workforce strategies must therefore address both JD-R pathways by strengthening resources to promote engagement and reducing strain to prevent burnout.

The rural context played a critical role in shaping these dynamics. Structural constraints such as chronic staffing shortages and limited specialist availability heightened vulnerability to burnout, while strong community ties and close patient–provider relationships may have functioned as unique job resources sustaining engagement. The absence of a direct association between COVID-19 caregiving and either outcome suggests that, by the seventh wave, COVID-related duties had become normalized through accumulated experience, improved infection-control confidence, and psychological adaptation.

Overall, this study offers actionable implications for nursing management and policy. Promoting job crafting opportunities and resilience-building initiatives may enhance engagement during future crises, while systematic mental health screening and accessible psychological support are essential to mitigate burnout. Importantly, rural healthcare systems require context-sensitive workforce strategies that address enduring structural challenges through staffing reinforcement, workload redistribution, and sustainable workforce development.

By clarifying the indirect and conditional pathways linking the COVID-19 care experience to work engagement and identifying distinct predictors of work engagement and burnout, this study advances understanding of nurse well-being under prolonged crisis conditions and supports the development of comprehensive, dual-track strategies to strengthen the nursing workforce during future public health emergencies.

## Data Availability

The datasets generated and/or analyzed during the current study are not publicly available due to ethical restrictions, but they are available from the corresponding author on reasonable request.

## Abbreviations

BAT-J: Burnout Assessment Tool (Japanese version)
ICU: Intensive care unit
JILPT: Japan Institute for Labor Policy and Training
JCS-J: Job Crafting Scale (Japanese version)
JD-R: Job Demands-Resources model
RS-14: Resilience Scale Short Version (Japanese version)
STROBE: Strengthening the Reporting of Observational Studies in Epidemiology
UWES-3: Utrecht Work Engagement Scale-3 (Japanese version)
WHO: World Health Organization
S-WHO-5-J: WHO-5 Well-Being Index, short version (Japanese version)
